# Metatranscriptomic Identification of Diverse and Divergent RNA Viruses in Green and Chlorarachniophyte Algae Cultures

**DOI:** 10.3390/v12101180

**Published:** 2020-10-19

**Authors:** Justine Charon, Vanessa Rossetto Marcelino, Richard Wetherbee, Heroen Verbruggen, Edward C. Holmes

**Affiliations:** 1 Marie Bashir Institute for Infectious Diseases and Biosecurity, School of Life and Environmental Sciences and School of Medical Sciences, The University of Sydney, Sydney, NSW 2006, Australia; justine.charon@sydney.edu.au (J.C.); vrmarcelino@gmail.com (V.R.M.); 2 Centre for Infectious Diseases and Microbiology, Westmead Institute for Medical Research, Westmead, NSW 2145, Australia; 3 School of BioSciences, University of Melbourne, Parkville, VIC 3010, Australia; richardw@unimelb.edu.au (R.W.); heroen@unimelb.edu.au (H.V.)

**Keywords:** algae viruses, protist viruses, RNA-dependent RNA polymerase, RNA virus metatranscriptomics, evolution, phylogeny

## Abstract

Our knowledge of the diversity and evolution of the virosphere will likely increase dramatically with the study of microbial eukaryotes, including the microalgae within which few RNA viruses have been documented. By combining total RNA sequencing with sequence and structural-based homology detection, we identified 18 novel RNA viruses in cultured samples from two major groups of microbial algae: the chlorophytes and the chlorarachniophytes. Most of the RNA viruses identified in the green algae class Ulvophyceae were related to the *Tombusviridae* and *Amalgaviridae* viral families commonly associated with land plants. This suggests that the evolutionary history of these viruses extends to divergence events between algae and land plants. Seven *Ostreobium* sp-associated viruses exhibited sequence similarity to the mitoviruses most commonly found in fungi, compatible with horizontal virus transfer between algae and fungi. We also document, for the first time, RNA viruses associated with chlorarachniophytes, including the first negative-sense (bunya-like) RNA virus in microalgae, as well as a distant homolog of the plant virus *Virgaviridae*, potentially signifying viral inheritance from the secondary chloroplast endosymbiosis that marked the origin of the chlorarachniophytes. More broadly, these data suggest that the scarcity of RNA viruses in algae results from limited investigation rather than their absence.

## 1. Introduction

Viruses are likely to infect every cellular organism and play fundamental roles in biosphere diversity, evolution, and ecology. Those studies of the global virosphere performed to date have revealed marked heterogeneities in virus composition. For example, while RNA viruses are commonplace in eukaryotes, they are less often found in bacteria and are yet to be conclusively identified in archaea. Rather, both the bacteria and archaea are dominated by DNA viruses [1,2]. It is unclear, however, whether such highly skewed virus distributions reflect fundamental biological, cellular or ecological factors of the hosts in question, or because RNA viruses in bacteria and archaea are often so divergent in sequence that they are difficult to detect using primary sequence comparisons alone.

The advent of “omics” technologies has fueled more intensive efforts to assess global viral diversity, especially in marine environments [3,4,5,6,7]. However, despite the substantial increase in virus sampling, our picture of the virosphere remains largely restricted to bacteria, some animal lineages and plants [8,9,10,11,12]. Clearly, such a sampling bias also impacts our understanding of the fundamental patterns and processes of virus evolution. A good example of this major knowledge bias are the unicellular eukaryotes, grouped under the term “protists”, and particularly the microalgae. Despite recent efforts to document the RNA virome of marine micro-organisms [2,13,14], to date only 61 viruses have been formally recognized in microalgae [15] comprising just 82 viral sequences [16]. This represents only 0.6% of the total 14,679 viral sequences listed on the Viral-Host database (release April 2020).

Since the first cultivation in 1979 [17,18], the isolation and characterization of algal viruses (phycoviruses) has largely focused on those with DNA genomes [15,19] (including 55 of the 82 algal virus sequences available at VirusHostdb, release April 2020). These include the well-known giant viruses, the majority of which (53%) have been described in the green algae (Chlorophyta). The DNA-dominated virome of green algae contrasts with those of their sister-group, the land plants [20], for which 60% of the 3590 viral reference entries are RNA viruses (i.e., the “*Riboviria*”; VirusHostdb, April 2020 release). Most (85%) of the 27 RNA viruses described from algae to date have been identified in diatoms [21]. Although limited in number, the algal RNA viruses characterized thus far display impressive diversity, belonging to the families *Totiviridae, Reoviridae, Marnaviridae, Endornaviridae, Flaviviridae, Narnaviridae* and *Alvernaviridae*. It is currently unclear, however, whether the seemingly differing distributions of DNA and RNA viruses reflect a major switch in virus composition that occurred during the expansion of land plants or is indicative of the inherent limitations in sampling and cultivation of both algae and their viruses, or the difficulties in identifying highly-divergent RNA viruses [22]. Indeed, because RNA viruses are the fastest evolving entities described [23], phylogenetic signal is rapidly lost over evolutionary time. Hence, it is possible that the low number of algal RNA viruses detected to date simply reflects the fact that they are highly divergent in sequence, even in the canonical RNA-dependent RNA polymerase (RdRp), and hence refractory to detection using primary sequence similarity. Importantly, protein structures are expected to be an order of magnitude more conserved than amino acid sequences [24]. As a consequence, the study of conserved secondary or tertiary structures could help identify distant homologies among RNA viruses [25,26], including novel viruses within the microalgae.

Given the phylogenetic diversity and array of genomic features characterized to date in algal RNA viruses (with linear, circular, segmented, nonsegmented, single-strand and double-strand genomes), as well as their wide range of habitats and ubiquity, it is reasonable to expect that microalgae will harbour an abundance of RNA viruses. In addition, 72,500 species of microalgae have been identified to date distributed across diverse branches of the eukaryotic phylogeny (in the TSAR (Telonemia, Stramenopiles, Alveolates, Rhizaria), Archaeplastida, Haptista, Cryptista and “excavates” supergroups [27,28,29]), with estimates of the true number of species in excess of 300,000 [30,31]. Microalgae constitute a primary food source in the marine and freshwater food chain. Together with the ancient (ca. 1.8 billion years) nature of eukaryotic algae and their involvement in secondary plastid endosymbiosis events involving many branches of the eukaryotic phylogeny, it has been proposed that algal viruses played a key role in the early evolution of eukaryote viruses [15,32]. Revealing the nature of RNA virus diversity in algae may therefore have major consequences for understanding the processes that have shaped long-term virus biodiversity and evolution.

We aimed to reveal more of the RNA virosphere in cultured samples of two clades of microalgae: (i) the green algae (Chlorophyta), that are part of the Archaeplastida eukaryotic supergroup, and (ii) the chlorarachniophytes, a lineage of Rhizaria that obtained a chloroplast through secondary endosymbiosis of a green alga [33]. To this end we performed an unbiased (i.e., bulk RNA-sequencing) meta-transcriptomic analysis, with an emphasis on detecting remote signals of homology in the RdRp, the gene hallmark of RNA viruses, using protein-profile based approaches. The comparison of the viromes of these two distant groups enabled us to address a number of fundamental evolutionary questions: (i) is the deep divergence between the two algae taxa also reflected in their RNA virome compositions? (ii) does their RNA virome provide evidence for complex evolutionary histories, including horizontal transfer events? (iii) is the RNA/DNA virus bias between algae and green plants an artefact of sampling or reflect a more fundamental biological division? More generally, we aimed to broaden our understanding of the biodiversity of the algal virosphere as this may have implications for understanding and managing the roles played by algae in global element cycling, climate forcing and biotechnology, and as reservoirs for genetic novelty [34,35].

## 2. Materials and Methods

### 2.1. Algal Cultures

Algal strains were isolated from marine sand (*Microrhizoidea pickettheapsiorum* [36]; *Kraftionema allantoideum* [37]; *Chlorarachnion reptans* and *Lotharella* sp. from the Wye River, Victoria, Australia) or coral skeletons (*Ostreobium* sp. HV05007, Kavieng, Papua New Guinea), or obtained from the NIVA Culture Collection of Algae (*Dolichomastix tenuilepis*, SCCPA strain K-0587). Cultures were maintained in K-enriched seawater medium (transferring every other week) at either 26 °C (*Ostreobium*) or 16 °C (all others) under cool white LED lights at 1 Photosynthetic active radiation (PAR) (*Ostreobium*) or 15 PAR (all others). Cultures were pelleted by centrifugation in falcon tubes and stored in RNAlater at −80 °C until RNA extraction.

### 2.2. Total RNA Extractions

For total RNA extractions, RNAlater was removed by low centrifugation, algal cells were disrupted using thaw/freezing cycles and bead beating (0.1–0.5 mm), and total RNA was extracted using the Qiagen ^®^ RNeasy Plant mini kit following the manufacturer’s instructions.

For the initial meta-transcriptomic screening, RNAs were pooled into three groups: (i) the chlorophyta *Dolichomastix tenuilepis* and *Microrhizoidea pickettheapsiorum* (Mamiellophyceae) and *Kraftionema allantoideum* (Ulvophyceae) were pooled into meta-transcriptome ‘ALG_1′; (ii) the ulvophyte *Ostreobium* sp. comprised ‘ALG_2′; and (iii) the two chlorarachniophytes *Chlorarachnion reptans* and *Lotharella* sp. were pooled into ‘ALG_3′ (Table 1).

### 2.3. Total RNA Sequencing

RNA quality was assessed and TruSeq stranded libraries were synthetized by the Australian Genome Research Facility (AGRF), using either (i) TruSeq stranded with a eukaryotic rRNA depletion step (RiboZero Gold kit, Illumina) for ALG_2, or (ii) the SMARTer Stranded Total RNA-Seq Kit v2—Pico Input Mammalian libraries (Takara Bio, Mountain View, CA, USA) for ALG_1 and ALG_3 (Table S1), due to the low amount of input RNAs in these libraries. The resulting libraries were sequenced on an Illumina HiSeq2500 (paired-end, 100bp) at the AGRF. Library descriptions and RNA-seq statistics are summarized in Table S1.

### 2.4. In Silico Processing of Meta-Transcriptomic Data

#### 2.4.1. Read Depletion and Contig Assembly

The RNA-seq data were first subjected to low-quality read and Illumina adapter filtering using the Trimmomatic v0.36 program [38]. Ribosomal RNA was depleted with the SortmeRNA v3.0.3 program [39] using the SILVA v32 database [40], which removed between 86 and 94% of the total unfiltered reads (Figure S1A/2). Read-depleted libraries were then de novo assembled using the Trinity v2.5.1 program [41] and contigs shorter than 200 nt were removed (the average length of contig assembly is shown in Figure S1B/2). Contig abundances were calculated from the RNA-seq data using the Expectation–Maximization (RSEM) v1.3.1 software [42] and expressed as the expected read counts. An analysis of the assembly quality was attempted by estimating the proportion of full-length transcripts in each library using the “analyze_blastPlus_topHit_coverage.pl” script available in Trinity package. Briefly, this analysis consists in aligning all the transcripts obtained after each de novo assembly against the SwissProt/UniProt database using BLASTx and extracting the number of proteins aligned depending on their level of coverage (percentage of the top-hit sequence).

#### 2.4.2. RNA Virus Detection Using BLASTx and BLASTn

The similarity of contigs to the current NCBI nucleotide (nt) and protein (nr) databases was determined using the BLASTn v2.2.30 and Diamond BLASTx v0.9.32 programs [43], respectively, employing 10^−10^ and 10^−05^ as e-value cut-offs and the more sensitive option in BLASTx. RNA virus-like sequences were also identified using BLASTx against all RdRp protein sequences available on NCBI/GenBank. False-positive signals for RNA viruses were removed by BLASTing RdRp-like sequences against the nr database and discarding sequences displaying a nonviral sequence as the best hit, based on BLASTx scores.

#### 2.4.3. RNA Virus Profile-Based Homology Detection

To detect especially diverse RdRp-based sequences, orphan contigs (i.e., those with no match in either the nr and nt databases) were compared to the Pfam RdRp-protein profiles ‘MitoVir_RdRp’ (PF05919), ‘Birna_RdRp’ (PF04197), ‘Viral_RdRp_C’ (PF17501), ‘RdRP_1′ (PF00680), ‘RdRP_2′ (PF00978), ‘RdRP_3′ (PF00998) and ‘RdRP_4′ (PF02123), as well as to the entire VOG profile database (http://vogdb.org) [44] using the HMMer3 v3.3 program [45]. To check for false-positive signals, these orphan sequences were submitted to the entire Pfam database using the same HMMer version and default parameters.

#### 2.4.4. D Protein Structure Prediction of RdRp-Like Contigs

To infer a structural model for the distant RdRp signals detected using profiles, sequences displaying a RdRp-like signal were subjected to the normal mode search of the Protein Homology/analogY Recognition Engine v 2.0 (Phyre2) web portal [46]. Briefly, this program first compares the submitted amino acid sequence to a curated nonredundant nr20 data set using HHblits [47]. It then converts the conserved secondary structure information as a query against known 3D-structures using HHsearch [48]. A final structural modeling step based on identified structural homologies is performed as described previously [46].

#### 2.4.5. RNA Virus Sequence Analysis and Annotation

Total non-rRNA reads were mapped onto RNA virus-like contigs using Bowtie2 v2.3.3.1 and heterogeneous coverage and potential mis-assemblies were manually resolved using Geneious v11.1.4 [49]. Open reading frames (ORFs) were first predicted using getORF from EMBOSS v6.6.0, in which ORFs were defined as regions that are free of stop codons (−find 0 option), although partial sequences (i.e., missing start or stop codons) were retained for analysis. Protein domains were annotated using the InterProscan software package from EMBL-EBI, using the InterPro consortium databases PROSITEpatterns v2019_11, PROSITEprofiles v2019_11, PRINTS v42.0, Pfam v33.1, PIRSF v3.10, TIGRFAM v15.0, SuperFamily, CDD v3.17 and PANTHER v14.1 (https://github.com/ebi-pf-team/interproscan).

#### 2.4.6. Revealing Host-Virus Associations

A challenge faced by all metagenomic studies is confidently assigning each viral sequence to a particular host in a given sample. We used algal cultures to minimize the number of potential additional cellular hosts. These cultures were, however, nonaxenic (i.e., cultures not purified from other contaminating organisms), with mainly bacteria present. To evaluate the possibility of additional microeukaryotic cells in the sample, we obtained taxonomic identification for contigs in the meta-transcriptome by aligning them to the NCBI nt database using the KMA aligner v1.2.11a and the CCMetagen program [50,51] v1.1.3. Contigs matching an entry in the nt database were displayed as Krona plots and classified based on their taxonomy (using high taxonomic levels for clarity).

#### 2.4.7. Phylogenetic Analysis

RdRp amino acid sequences were aligned using the L-INS-I algorithm and default parameters in the MAFFT program v7.402 [52] and trimmed with TrimAI v1.4.1 (automated1 model). Maximum likelihood phylogenetic trees were then estimated using IQ-TREE v2.0-rc1, employing ModelFinder to obtain the best-fit model of amino acid substitution in each case, with nodal support assessed using 1000 bootstrap replicates and 1000 replicates of SH-like approximate likelihood ratio test (SH-aLRT) [53,54]. For each tree, reference genomes and corresponding RdRp sequences were retrieved from the NCBI viral genome resource (https://www.ncbi.nlm.nih.gov/genome/viruses/). To depict the evolutionary relationships of the newly discovered viruses as meaningfully as possible, the closest unclassified BLASTx homologs were used in the phylogenetic analysis. This resulted in alignments of 237, 76, 198, 558 and 325 RdRp protein sequences for Amalga-like, Mito-like, Tombus-like, Virga-like and Bunya-like virus groups, respectively.

### 2.5. RT-PCR Validation

Viral contigs were validated experimentally and associated to individual algal sample using Reverse Transcription (SuperScript ™ IV reverse transcriptase – Invitrogen, Carlsbad, CA, USA ™) followed by PCR (Platinum ™ SuperFi ™ DNA polymerase – Invitrogen ™), with specific primer sets for each contig. The *rbc*L and *tuf*A marker genes were used as PCR positive controls using sets of primers designed in [55]. All primers and PCR conditions used in this study are described in Table S2.

### 2.6. Data Availability

The libraries sequenced here are available at the Sequence Read Archive (SRA) under BioProject PRJNA668187. The consensus sequences of the all novel viruses identified here have been submitted to GenBank and assigned accession numbers MW086576-MW086593.

## 3. Results

### 3.1. The RNA Viromes of Two Divergent Groups of Microalgae

**Figure 1:**
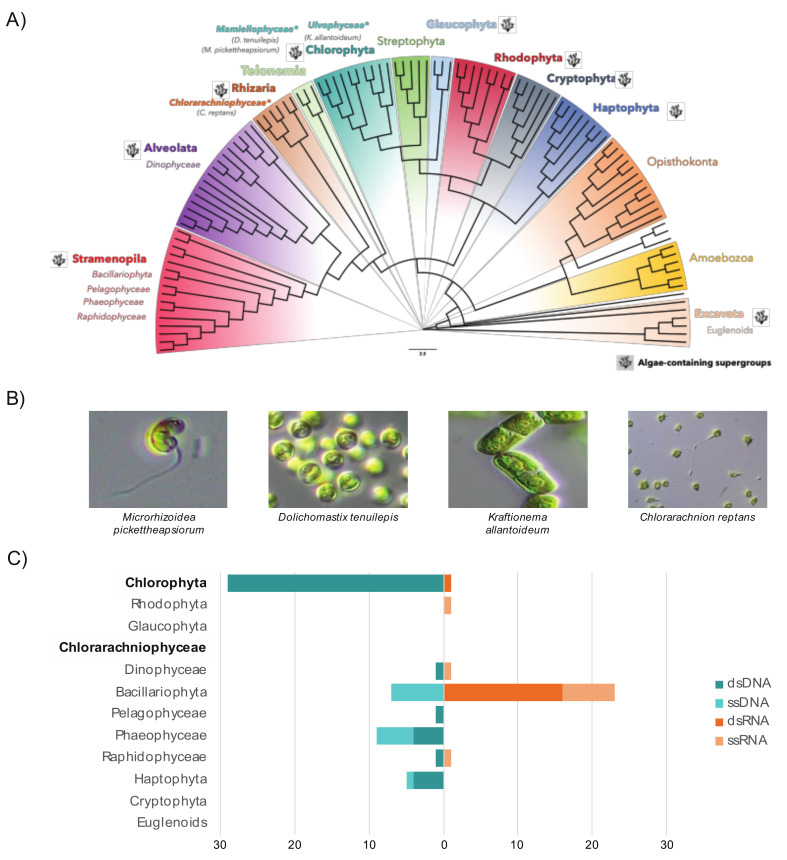
The enormous diversity of algae contrasts with their poorly characterized viromes. (**A**) Representation of algae supergroups among the diversity of eukaryotes (latest eukaryotic classification retrieved from [29]). The phylogenetic tree was adapted from [56]. Pictures illustrate some of the samples used in this study and corresponding clades are marked with “*”. (**B**) Pictures of algae cultures used in this study. (**C**) The current extent of the microalgae virosphere. The viral sequence counts for each virus class (DNA or RNA, single-stranded or double-stranded) were retrieved from VirusHostdb [16] according to 11 major eukaryotic algae lineages. Microalgal lineages investigated in this study are highlighted in bold.

Our aim was to determine the RNA viromes of six microalgae cultures from six different algal species classified into two highly phylogenetically distinct algal clades: the chlorarachniophytes (Rhizaria) and the green algae (Chlorophyta, Archaeplastida) (Figure 1A,B). To the best of our knowledge, this is the first identification of viruses in samples from the Chlorarachniophyceae (Figure 1C) [15].

While our limited understanding of chlorarachniophyte viruses can be explained by the small number of species characterized to date (only 15 in Algaebase database), the Ulvophyceae is an abundant and diverse algal lineage in existence since the late Proterozoic and comprises at least 1933 species [57]. It contains a wide range of morphologies from unicellular benthic algae to large seaweeds [58] and its representatives commonly occur in marine, terrestrial and freshwater habitats [20]. We therefore performed RNA sequencing (meta-transcriptomics) on six microalgal species belonging to both the green algae (classes Ulvophyceae and Mamiellophyceae) and chlorarachniophytes (Table 1).

Because of variable RNA quality and quantity, fewer nonrRNA reads were obtained for the ALG_1 and ALG_3 libraries. This may in large part explain the limited length of contigs, the reduced number of estimated full-length transcripts, and ultimately the lower number of viral sequences, compared to ALG_2 (Figure S3).

In total, we identified 18 new putative viral sequences using a standard sequence similarity search among the three libraries. These largely comprised viruses with double-stranded (ds) RNA or single-stranded positive-sense (ssRNA(+)) genomes (Table 2). However, a divergent bunya-like partial sequence was also retrieved from *Chlorarachnion reptans* and may constitute the first negative-sense (ssRNA(−−−)) virus identified in microalgae. Importantly, the presence of these viruses was validated by RT-PCR on each total extracted RNA (Figure S1). In each case these viruses exhibited very low levels of sequence similarity to existing RdRp amino acid sequences, with sequence identities ranging from only 27 to 38%. With the exception of Virga-like bellevilovirus, Bunya-like bridouvirus and Amalga-like boulavirus for which partial sequences have been retrieved, the length and genomic organization (ORF numbers, predicted protein length, etc.) of all of the new viruses described in this study are similar to the well-annotated full-length genomes of reference homologs. It is therefore likely that they correspond to full-length genomes. In addition, the lack of frameshifts and premature stop codons means these sequences are very likely true (exogenous) viruses rather than endogenous viral elements (EVEs) inserted into host genomes.

Eight of the viruses identified in *Ostreobium* sp. fell within the *Narnaviridae* or *Tombusviridae.* In contrast, five viral sequences from *Ostreobium* sp. and *K. allantoideum* do not fit into defined taxonomic groups and were instead related to the broad set of ‘partiti-like’ viruses that comprise the *Partitiviridae*, *Totiviridae* and *Amalgaviridae*. Finally, more divergent but detectable sequence similarities to the *Virgaviridae* (+ssRNA) were obtained for samples from the chlorarachniophyte library.

### 3.2. Detection of Divergent Viruses Using Protein Structural Data

**Figure 2:**
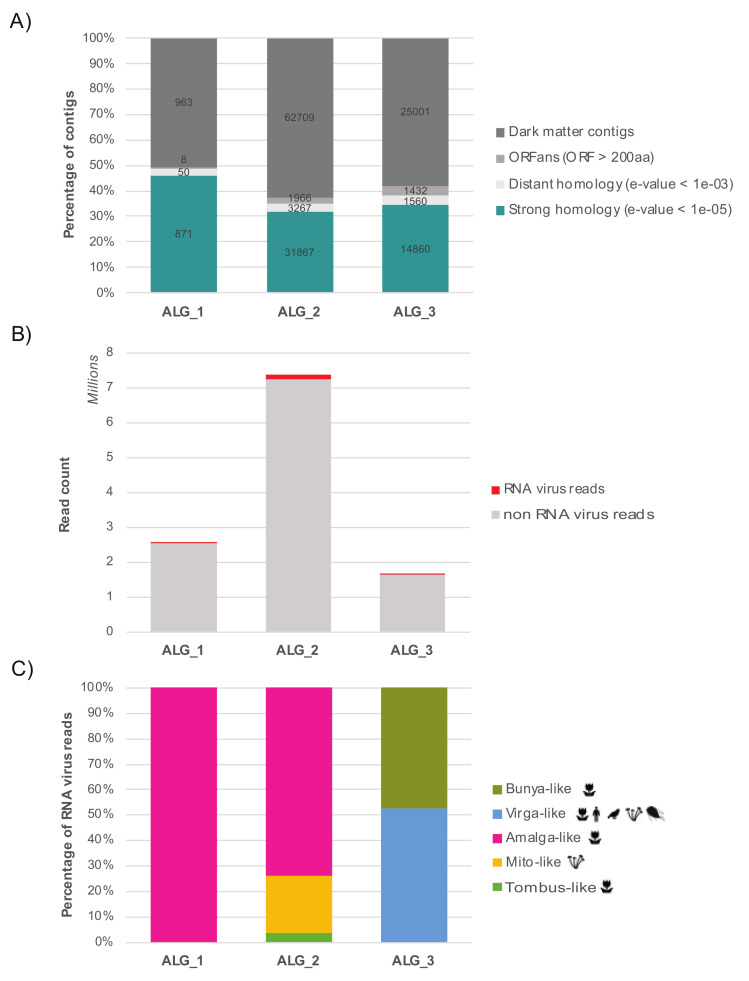
Abundance of unknown and RNA virus-like contigs detected in the algal libraries. (**A**) Percentage of nonassigned contigs. For clarity, numbers are normalized as the percentage of total contigs (actual contig numbers are indicated in bold). Blue: number of contigs showing strong sequence similarity to the nr database (e-value < 10^−05^); light grey: contigs showing weak sequence similarity to the nr database (e-values 10^−05^ to 10^−03^); middle-dark grey: contigs with no sequence similarity detected by BLASTx/BLASTp but predicted to encode one or more ORFs longer than 200 amino acids (600 nt); dark grey: genomic ‘dark matter’ - contigs without any signal detected or any long ORFs encoded. (**B**) Total number of RNA virus reads per total number of non-rRNA reads in each library. (**C**) Distribution of RNA virus diversity in the three libraries and percentage of RNA virus reads associated with each viral super-clade. The host range is represented for each viral clade.

An additional attempt to detect even more divergent RNA viruses was conducted was using protein structure. In particular, it is possible that highly divergent viruses are part of the unknown orphan sequences (i.e., contigs with no match in nt/nr databases, or the ‘dark matter’) that comprise between 50–60% of total contigs obtained in this study (Figure 2A).

Accordingly, we attempted to detect evolutionary-conserved features of protein structural and functional motifs in orphan sequences that encode unknown ORFs, using a cut-off of 200 amino acids (600 nucleotides): we chose this size because it is shorter than most RdRps [59] yet should be long enough to make evolutionary inferences. The corresponding translated ORFs were compared to protein profiles from the PFAM RdRp clan and the VOG databases using the hidden-Markov model-based HMMer3 program. To help exclude false-positives, all positive hits were compared to the entire PFAM database. This resulted in the identification of three nonphage contigs that displayed homology to the RNA virus RdRp: ALG_2_DN19089, ALG_2_DN594 and ALG_3_DN34624 (Table 3).

To manually assess the level of confidence of the RdRp signal detected in the HMM comparisons, the protein sequences of the three RdRp candidates were aligned to amino acid sequences retrieved from the RdRp_C, RdRp_4 and RdRp_1 PFAM profiles. The ALG_2_DN594 contig displayed similarities with the RdRp_C profile that represents the C-terminal of the RdRp (Protein A) found in alphanodaviruses. Unfortunately, this C-terminal region lacks the key functional motifs usually associated with the RdRp, preventing us from definitively establishing the ALG_2_DN594 contig as a true RNA virus. Similarly, the ALG_3_DN34624 alignment with the viral RdRp sequences that comprise the PFAM RdRp_4 profile (PF02123) does not show conservation of the crucial functional residues at the A, B and C-motifs within the RdRp, particularly the canonical motif C that is normally GDD, yet GFD in ALG_3 contig (Figure S2B): strikingly, the GFD motif is absent from an alignment of 4627 viral RdRp sequences [60]. Whether this reflects a newly identified functional motif remains to be determined, but we cannot safely conclude that ALG_3_DN34624 encodes a viral RdRp. In contrast, the ALG_2_DN19089-encoded ORF shared motifs with RdRp_1 profile (PF00680), including motif A at positions 437–442, motif B at positions 507–517 and a GDD motif C at positions 557–559 of the RdRp alignment (Figure S2A). Because of the presence of these functional motifs, ALG_2_DN19089 can be confidently considered as a true RdRp-encoding contig and will be referred to here as ‘Partiti-like adriusvirus’. Interestingly, this contig also revealed significant similarity to some eukaryotic chloroplast-associated double-stranded RNA replicons (BDRC) obtained from the green algae species *Bryopsis cinicola* [61] (Table 2). It is therefore likely that these BDRC dsRNA *Bryopsis*-replicons in fact represent viral RdRp sequences [22], and we treat them as such in this study.

An additional BLASTx comparison using this divergent Partiti-like RdRp as a reference identified two other BDRC-like contigs in the *Ostreobium* sp. data set—ALG_2_DN19300 and ALG_2_DN19436 (Table 2). Along with the Partiti-like adriusvirus, these two additional sequences were both validated by RT-PCR (Figure S1) and are listed in the viral contig table as ‘Partiti-like lacotivirus’ and ‘Partiti-like alassinovirus’, respectively (Table 2).

The remaining hits from the RdRp-profile analysis—ALG_2_DN594_c0_g2_i1_len711 and ALG_3_DN34624_c0_g1_i1_len2077—were used in a Phyre2 protein structural analysis. However, this revealed no confident identification of a viral RdRp (i.e., the confidence levels of structural models obtained were <90%).

### 3.3. Relative Abundance and Prevalence of RNA Viruses in the Samples

Relative virus abundance varied between libraries and viruses of the same family in the *Ostreobium* sp. culture, with viral-like sequences constituting between 0.01 (considered as average abundance) and 1.2% (considered as very high abundance) of the total non-rRNA reads (Table 2, Figure 2). Each virus described was identified in only one of the cultures sequenced (Figure S1). However, intersample BLAST-comparisons revealed similarity between the partiti-like sequence identified in the *K. allantoideum* and *Ostreobium* samples (30% amino acid identity). A nonannotated ORF from a *K. allantoideum* contig, ALG_1_DN2506, aligned with the N-terminal ORF of *Ostreobium* sp. amalga-like virus contigs that potentially encode the virus coat protein, but the high level of divergence prevented us from performing any phylogenetic analysis of proteins other than the RdRp. Because of their co-occurrence in *K. allantoideum* (1.1 and 1.2 PCR, Figure S1) and their similar abundance levels, it is likely that ALG_1 DN2506 and ALG_1 DN2691 (referred as ‘Amalga-like boulavirus’, Table 2) contigs are part of the same genome. Unfortunately, the poor quality of RNAs and the resulting high degree of fragmentation obtained in the ALG_1 RNA-seq library did not allow us to resolve this question.

Notably, a large majority of the new RNA viruses reported here come from the ulvophyte *Ostreobium* sp. clonal culture, although this may in part result from differences in RNA quality and sequencing rather than a true biological difference in RNA virome composition and diversity (Figure S3). Difficulties in detecting highly divergent viral sequences, especially in poorly characterized and distant clades such as the chlorarachniophytes, may also contribute to the different numbers of viruses observed between libraries.

### 3.4. Detection of Possible Secondary Hosts

As the algal cultures analyzed here were not axenic, we assessed the diversity and relative abundance of other potential eukaryotic organisms in these samples. Indeed, algae cultures are commonly co-cultured with bacteria, fungi and other endosymbiotic algae [62]. The rRNA depletion performed during the RNA-Seq library preparation prevented us from using standard 16S/18S profiling. We therefore evaluated the presence of other eukaryotes in the samples using CCMetagen [50]. According to the Krona plots obtained for each library, cultivated algae were, as expected, the dominant organism found in the samples, representing between 79–99% of all assigned contigs (Files S1–S3). Nevertheless, a small proportion (2–8%) of contigs from ALG_1 and ALG_3 were assigned to dinoflagellates and *Cyanophora* algae (Files S1 and S3). Although sequences assigned to *Lingulodinium polyedrum* potentially result from GenBank mis-annotation and were likely of bacterial origin, the *Coolia malayensis* (Dinophyceae), *Amphidinium sp.* (Dinophyceae) and *Cyanophora paradoxa* (Glaucophyta) associated sequences likely constitute true assignments. We therefore suspect that these additional micro-eukaryotic transcripts may have arisen from cross-contamination with additional algae samples that were extracted and prepared at the same time and sequenced in the same run. Importantly, however, none of the viruses identified in the three libraries studied here could be detected in the transcriptomes of co-processed Dinophyceae and Glaucophyta cultures, suggesting that these low-abundance contaminants are not the hosts of the viruses reported here. In addition, a minor portion of the contigs in ALG_2 and ALG_9 was assigned to bacterial species (0.3% and 5%, respectively; Supplementary Files S2 and S3). To prevent any misinterpretation, particular care was taken to remove bacteriophage-like signals from the final virus-like sequence files.

### 3.5. Phylogenetic Analysis of the Newly Identified Viruses

#### 3.5.1. Partiti-Like dsRNA Viruses

**Figure 3:**
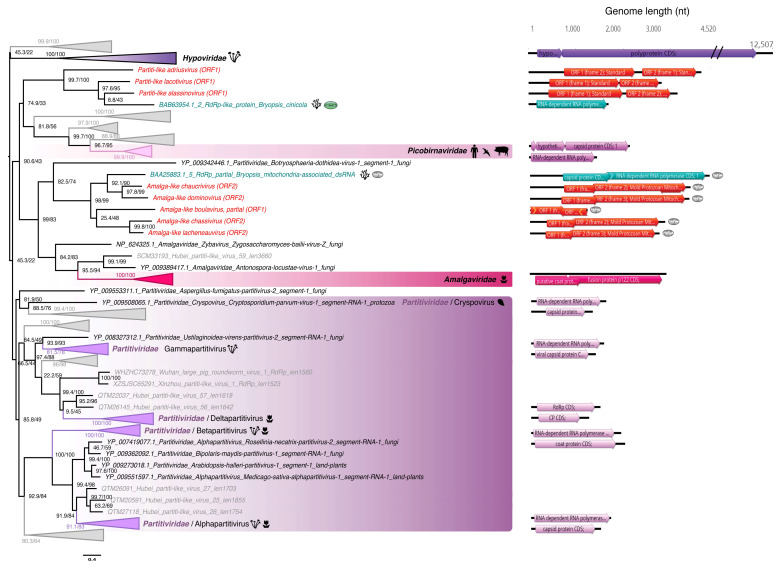
RdRp phylogeny of the newly identified chlorophyte viruses among the *Amalgaviridae*, *Partitiviridae*, *Picobirnaviridae* and *Hypoviridae*. Sequences identified in this study are labeled in red. Unclassified sequences from [65] are highlighted in grey. For clarity, some families and genera have been collapsed. Left, phylogenetic tree estimated using IQ-Tree with bootstrap replicates and SH-aLTR set to 1000 (values in parenthesis). Right, genomic organization of both viral genomes identified in this study (red) and representative species of each major family and genus used in the phylogeny (*Cryphonectria hypovirus 2*—*Hypoviridae*; *Chicken picobirnavirus*—*Picobirnaviridae*; *Southern tomato virus*—*Amalgaviridae*; *Cryptosporidium parvum virus 1*—*Cryspovirus*; *Discula destructiva virus 1*—*Gammapartitivirus*; *Figure cryptic virus*—*Deltapartitivirus*; *Ceratocystis resinifera partitivirus*—*Betapartitivirus*; *White clover cryptic virus 1*—*Alphapartitivirus*. The tree is mid-pointed rooted and branch lengths are scaled according to the number of amino acid substitutions per site.

Eight of the newly described viral sequences exhibited RdRp amino acid sequence similarity to Partiti-like viruses (i.e., relatives of the *Partitiviridae*) and found at various levels of abundance (Table 2). Based on phylogenetic studies, five of the viral-like sequences are close to those from the *Amalgaviridae* (named after their mosaic status comprising both a *Partitiviridae*-like RdRp and the dicistronic and monopartite genomic organization of the *Totiviridae* [63]) and form a clade with the *Bryopsis* mitochondria-associated dsRNA (BDRM), although they share only 28–32% sequence identity (Table 2, Figure 3). BDRM was first described as a dsRNA associated with mitochondria in *Bryopsis cinicola* macroalgae [64] and later classified as a virus by the International Committee on Taxonomy of Viruses (ICTV). Like *Ostreobium* and *Kraftionema*, *Bryopsis* belongs to the class Ulvophyceae, and it seems likely that all these five newly identified Amalga-like viral sequences (Figure 3) form an Ulvophyceae-infecting viral clade.

**Figure 4:**
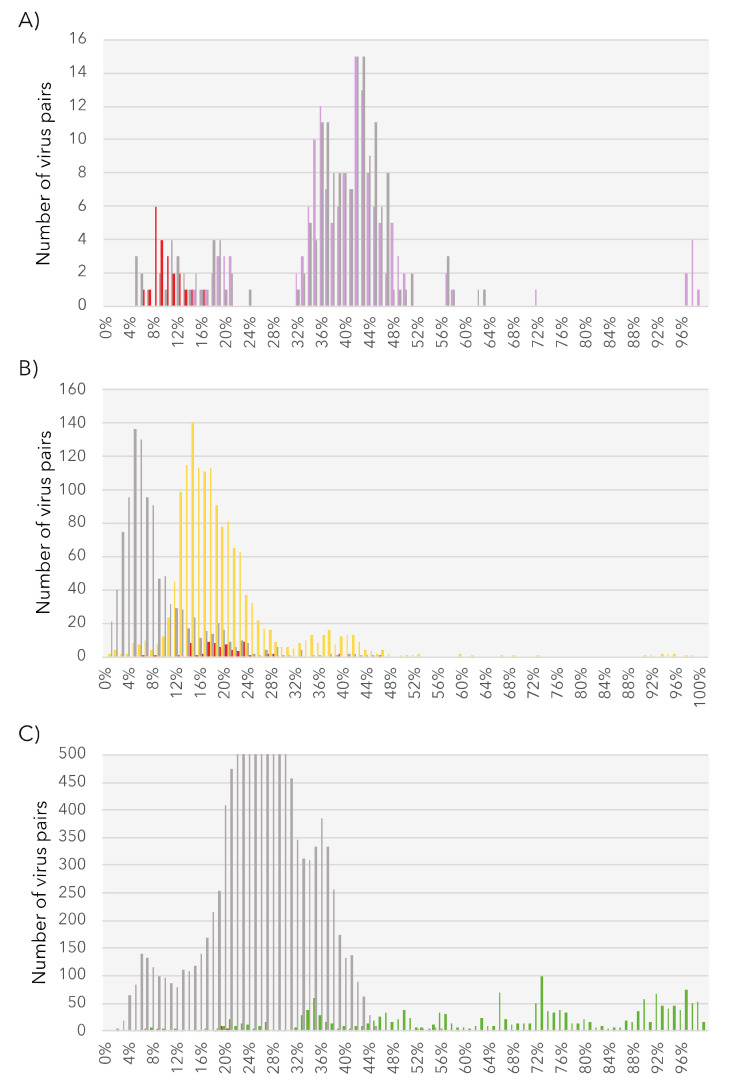
Genome pairwise identity distributions of the new algal viral sequences. The level of pairwise identity between the newly identified viruses and existing members of each viral family are represented in red. (**A**) Intergenus (grey) and intra-genus (purple) identity levels within the *Amalgaviridae*. **(B**) Intergenus (grey) and intra-genus (yellow) identity levels within the *Narnaviridae*. (**C**) Intergenus (grey) and intra-genus (green) identity levels within the *Tombusviridae*.

Given the level of RdRp pairwise identity between these sequences (Table S3, top) we assumed that each constituted a new species. To perform a preliminary taxonomic assessment, we used the PAirwise Sequence Comparison (PASC) tool available at the NCBI [66]. Each of these five new BDRM-like viral genome sequences were compared with the *Amalgaviridae* full-length genomes available. The closest matches to existing *Amalgaviridae* sequences were retrieved for each newly discovered virus, and the resulting pairwise identity distributions compared with those observed within and between *Amalgaviridae* genera (Figure 4A). While this analysis indicates that these newly identified virus sequences are not part of any existing *Amalgaviridae* genus (Figure 4A), whether they can be considered as a new genus within the *Amalgaviridae*, or even a new family, is currently unclear and will require formal taxonomic assessment by the ICTV.

Interestingly, if these Amalga-like sequences are translated into amino acids using the protozoan mitochondrial code they display the same genomic organization as BDRM, encoding two overlapping ORFs: the 5′ one encoding a hypothetical protein and the other encoding a replicase through a -1 ribosomal frameshift [67] (Figure S4). However, it is unclear if these sequences should be translated using the standard cytoplasmic code, and such sub-cellular localization remains to be validated. It is also notable that the two closest homologs of BDRM, the Amalga-like dominovirus and Amalga-like chaucrivirus, also contain the GGAUUUU ribosomal-1 frameshift motif and the two encoded ORFs could plausibly be translated in this manner (Figure S4).

The length and two-ORF encoding genomic structure of the BDRM-like sequences generally correspond to genomic features of the amalgaviruses (Figure 3, right). Despite a lack of detectable sequence similarity at both the sequence (BLASTx) and structural levels (i.e., the Phyre2 analysis), the second ORFs predicted in these amalgavirus-like sequences are expected to encode a CP-like protein, even if the involvement of this potential CP in encapsidation remains unclear [68].

**Figure 5:**
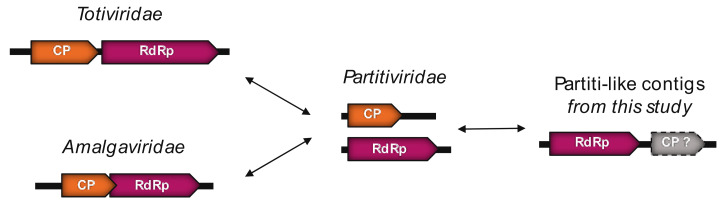
Genomic organization of the *Partitiviridae*, *Totiviridae* and *Amalgaviridae*. Possible evolutionary scenarios for the BDRC-like contigs observed in *Ostreobium* sp.

The three BDRC-like sequences identified from *Ostreobium* sp. also cluster with the Partiti-like viruses (Figure 3, Table 2) and can be classified as three different species after applying the commonly-used 90% RdRp percentage identity species demarcation criteria (Table S3, Middle). Notably, the genomic organization of these BDRC-like contigs seems “inverted” compared to members of the *Totiviridae* and *Amalgaviridae*; that is, a first ORF encoding a CP protein is followed by a second that represents the RdRp (Figure 5).

Indeed, the RdRp encoded by the Partiti-like ALG_2 contigs is close to the 5′ extremity, followed by a second ORF. This second ORF could potentially encode a CP, although functional annotation could not be achieved due to the high level of sequence divergence.

#### 3.5.2. Mitovirus-Like ssRNA(+) Viruses

**Figure 6:**
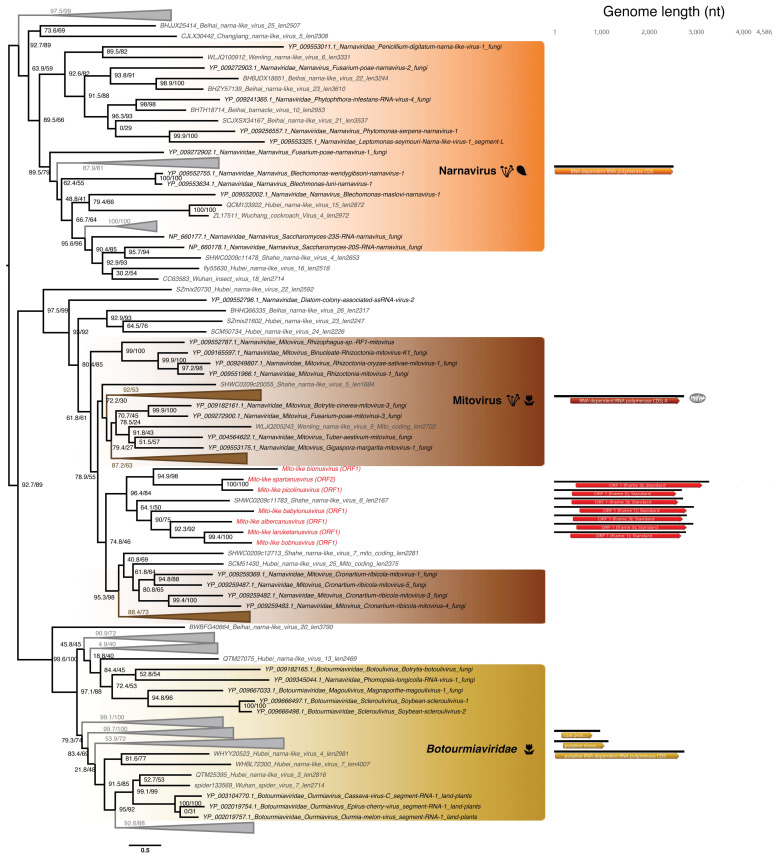
Phylogeny of the *Narnaviridae*-*Botourmiaviridae* group based on the RdRp. Newly discovered viruses from *Ostreobium* sp. are highlighted in red. RdRp sequences from unassigned RNA virus retrieved from [65] are marked in grey. Left, phylogenetic tree estimated using IQ-Tree with bootstrap replicates and SH-aLTR set to 1000 (values in parenthesis). Right, genomic organization of both viral genomes identified in this study (red) and representative species of each major family and genus used in the phylogeny (*Cassava virus C—Botourmiaviridae*; *Saccharomyces 23S RNA narnavirus—Narnavirus* genus; *Chenopodium quinoa mitovirus 1 Mitovirus* genus). Annotations of *Cassava virus C* coding sequences: RdRp (Segment I); Putative movement protein (Segment II); Coat protein (Segment III). Branch lengths are scaled according to the number of amino acid substitutions per site.

Seven viral sequences from *Ostreobium* sp. clustered in the *Narnaviridae*, forming a clade within the genus *Mitovirus* (Figure 6). With their single ORF likely encoding an RdRp, and a genome length of ~3000 nt that is typical of the *Narnaviridae* (Figure 6, right), and the relatively high levels of abundances (Table 2), these viral sequences very likely constitute replicating viruses and thus represent a newly-described clade of protist-associated mitoviruses potentially restricted to green microalgae. The closest relative virus identified, Shahe Narna-like virus 6, was isolated from freshwater small planktonic crustaceans (*Daphnia magna*, *Daphnia carinata* and *Moina macrocopa*) belonging to the order Cladocera [65]. These “grazer” animals feed on marine microorganisms including microalgae, and it is therefore possible that Shahe Narna-like virus 6 in fact infects ingested-algae rather than arthropod host, in a similar manner to other members of the *Narnaviridae*.

Based on the 50% RdRp sequence identity used as a species demarcation criterion in the *Narnaviridae* (ICTV report 2009), we identified seven new mito-like viral species (Table S3, bottom). To place of these new species among the *Narnaviridae* we performed a PASC analysis using the putative full-length genomes from the seven new viral species. This revealed that the identity levels of the new sequences fell in the range expected of intra-genus diversity (Figure 4B). We therefore propose the existence of a new subgroup of mitoviruses, comprising these seven new species as well as the Shahe Narna-like virus 6 (Figure 6). Whether this clade is associated with mitochondria is currently unclear, and predicted ORFs were obtained using both standard and mitochondrial genetic codes.

#### 3.5.3. *Tombusviridae*-Like ss(+)RNA Viruses

**Figure 7:**
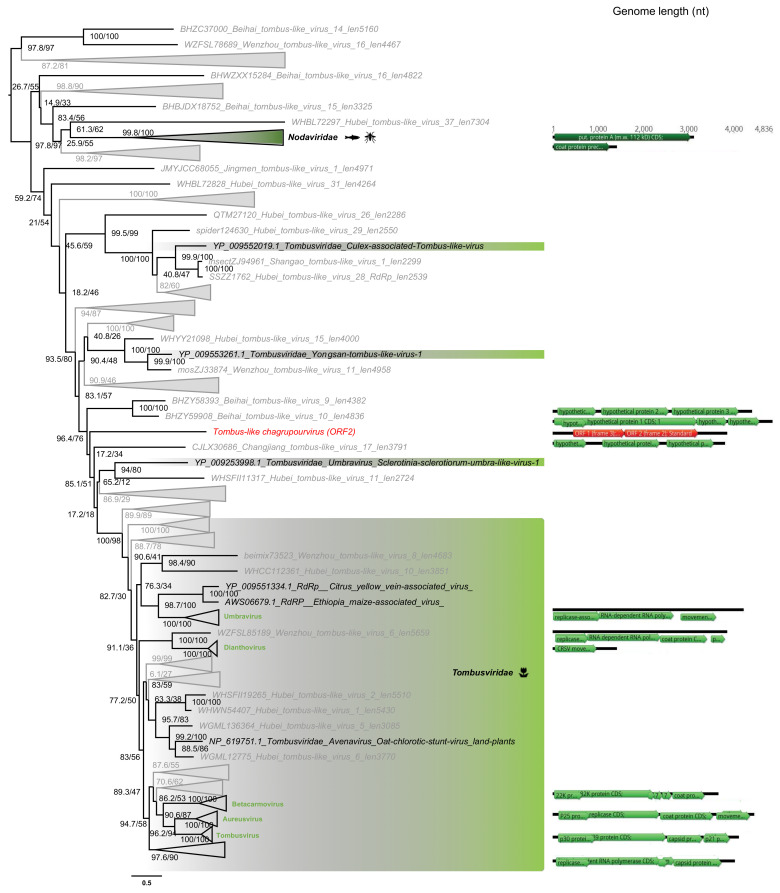
Phylogeny of the *Tombusviridae* RdRp. The tombus-like sequence identified in this study is labeled in red. Unclassified sequences from [65] are highlighted in grey. For clarity, some families and genera have been collapsed. Left, phylogenetic tree estimated using IQ-Tree with bootstrap replicates and SH-aLTR set to 1000 (values in parenthesis). Right, genomic organizations of the new viruses as well as their closest homologs and representative species from each family/genus as follows: *Black beetle virus* (*Nodaviridae*); *Carrot mottle virus* (*Dianthovirus*); *Carnation ringspot virus* (Dianthovirus); *Beet black scorch virus* (*Betanecrovirus*); *Cucumber leaf spot virus* (*Aureusvirus*); *Maize necrotic streak virus* (*Tombusvirus*); *Carnation mottle virus* (*Alphacarmovirus*). The tree is mid-pointed rooted and branch lengths are scaled according to the number of amino acid substitutions per site.

One sequence from *Ostreobium* sp., the Tombus-like chagrupourvirus, exhibited similarity to members of the *Tombusviridae* family of ssRNA(+) viruses (Figure 7), grouping with viruses previously identified as infecting plant or plant pathogenic fungi. This could again illustrate a shared evolutionary history between green algae and land plants, and that horizontal virus transfers can occur between plants and their pathogenic fungi. Of note is that the closest relative of Tombus-like chagrupourvirus documented to date, Hubei-Tombus-like virus 12, was isolated from freshwater animals (mollusca *Nodularia douglasiae)* [65]. According to the lack of distinguishable animal-related contigs in the *Ostreobium* sp. sample (File S2), and the average abundance level associated to this virus (0.7% of total non rRNA reads, Table 2), we assume this Hubei-Tombus-like virus 12 may, together with the newly tombus-like sequence identified here, constitute a new clade of green algae-infecting viruses.

The 3.8 kb genome length of the Tombus-like chagrupourvirus is similar to those commonly observed in *Tombusviridae* and their relatives (Figure 7, right), suggesting that it comprises a full-length genome for this virus. This putative full-length genome sequence was compared to *Tombusviridae* reference genomic sequences using PASC to assess its taxonomic position (Figure 4C). Accordingly, the *Ostreobium*-associated tombus-like sequence could constitute a new *Tombusviridae* genus.

#### 3.5.4. *Virgaviridae*-Like ssRNA(+) Viruses

**Figure 8:**
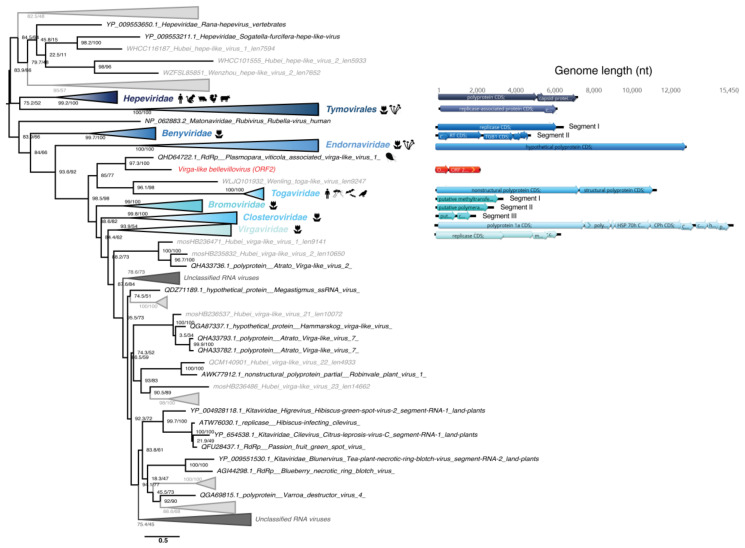
Phylogeny of the Hepe-Virga group RdRp. The hepe-virga-like sequence identified in this study is labeled in red. Unclassified sequences from [65] are highlighted in grey. For clarity, some families or genera have been collapsed. Left, RdRp-based phylogenetic tree obtained using IQ-tree with bootstrap replicates and SH-aLTR set to 1000 (values in parenthesis). Right, genomic organizations of the new viruses as well as closest homologs and representative species from each family/genus as follows: *Orthohepevirus A* (*Hepeviridae*); *Poinsettia mosaic virus* (order *Tymovirales*); *Wheat stripe mosaic virus* (*Benyviridae*); *Diatom colony associated dsRNA virus 15* (*Endornaviridae*); *Cabassou virus* (*Togaviridae*); *Apple mosaic virus* (*Bromoviridae*); *Mint virus 1* (*Closteroviridae*); *Cucumber mottle virus* (*Virgaviridae*). The tree is mid-pointed rooted and branch lengths are scaled according to the number of amino acid substitutions per site.

One sequence identified in the *Chlorarachnion reptans* culture displayed detectable sequence similarity to the *Virgaviridae*-like RdRp supergroup (Figure 8) and is present at average abundance in the library (0.01% of all non-rRNA reads). The family *Virgaviridae* comprise ssRNA(+) viruses traditionally associated with plants and display diverse genomic organizations. The short length of the Virga-like bellevillovirus associated with chlorarachniophytes indicates that this sequence likely comprises a partial genome sequence only. Moreover, the multi-segment structure of the closest relatives suggests that the partial genome recovered in ALG_3 could also contain additional segments not yet identified. Although further host confirmation is required, this newly described RNA virus-like sequence would constitute the first algae virus from the Hepe-Virga group.

#### 3.5.5. Bunyavirales-Like ss(-)RNA Viruses

**Figure 9:**
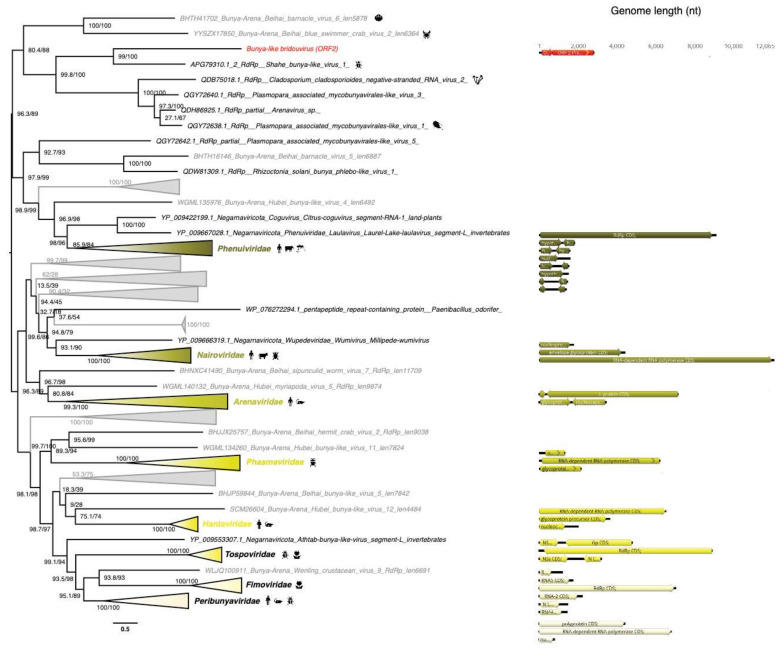
Phylogeny of the *Bunyavirales* RdRp. The bunya-like sequence identified in this study is labeled in red. Unclassified sequences from [65] are highlighted in grey. For clarity, some families and genera have been collapsed. Left, phylogenetic tree estimated using IQ-Tree with bootstrap replicates and SH-aLTR set to 1000 (values in parenthesis). Right, genomic organizations of the new viruses as well as their closest homologs and representative species from each family/genus as follows: *Melon chlorotic spot virus* (*Phenuiviridae*); *Yogue virus* (*Nairoviridae*); *Latino mammarenavirus* (*Arenaviridae*); *Seattle orthophasmavirus* (*Phasmaviridae*); *Melon yellow spot virus* (*Tospoviridae*); *Fig mosaic emaravirus* (*Fimoviridae*); *Tataguine orthobunyavirus* (*Peribunyaviridae*). The tree is mid-pointed rooted and branch lengths are scaled according to the number of amino acid substitutions per site.

A partial viral genome, denoted Bunya-like bridouvirus, encoding a RdRp-like signal was identified in the *Chlorarachnion reptans* sample at an abundance of 0.01% of total non rRNA reads. However, this sequence is highly divergent and cannot be formally assigned to any previously described viral family. Despite this, it is striking that the sequence clusters with a Bunya-Arena-like virus, Shahe bunya-like virus 1 (Figure 9) previously identified in diverse Freshwater small planktonic crustaceans (*Daphnia magna*, *Daphnia carinata* and *Moina macrocopa*) [65] that typically feed on algae. Our phylogenetic analysis places this sequence within the diversity of the order *Bunyavirales* (Figure 9). In addition to the freshwater organism-associated viruses identified in [65], this contig clusters with several bunya-like unclassified negative-strand viruses isolated from the fungi *Cladosporium cladosporioides* and the oomycete *Plasmopara viticola* (Figure 9) that are both plant pathogens. The multi-segment structure of the closest classified family, the *Phenuiviridae*, suggests that additional segments associated with the partial Bunya-like bridouvirus genome may exist in *C. reptans*.

## 4. Discussion

We aimed to better characterize the RNA virus diversity in two major algal lineages, the chlorarachniophytes and the ulvophytes, for which no RNA viruses had previously been reported. Our investigation of the RNA virus diversity in samples from six microalgae species led to the identification of 18 new and divergent RNA viruses, although with clear homology to five established viral families. While an unequivocal host assignment cannot be formally established on these metagenomic data alone, that the algae studied were the dominant host species in the metagenomic sequencing data, in one case (*Ostreobium* sp.) representing 99% of all assigned contigs, makes it likely that most if not all of these 18 viruses infect algae hosts. In addition, we identified a number of narnaviruses, a group previously observed in algae [69,70], and our observation of a *Bunyavirales*-like sequence is similarly in accord with a study that presented evidence for the presence of bunya/phlebo-like viruses in brown algae [71]. As such, the apparent domination of DNA viruses in microalgae at least partially reflects major sampling biases. The concept that there is a potentially large dark matter of algal viruses is further supported by the high proportion of unassigned contigs observed: we speculate that these likely contain a nonneglectable number of highly divergent viral reads.

### 4.1. RNA Virome Similarities between Green Algae and Land Plants

Among the 18 novel RNA virus species described here, seven of those detected in the green algae *Ostreobium* sp. and *K. allantoideum* were seemingly related to the *Tombusviridae* and *Amalgaviridae* families of plant RNA viruses. Such similarities in RNA virome composition between green algae and land plants are consistent with previous analyses based on the Plant Genome project transcriptomic data that identified partitivirus-like signals in Chlorophyte algae [22]. However, the very limited sequence similarity among these viral families strongly suggests an ancient divergence among them, perhaps even before the chlorophyte-streptophyte split some 850–1100 million years ago (Ma) [57]. The close link between land plant and green algae RNA virosphere is further supported by the recent observation that plant viruses are able to infect nonvascular plants such as mosses and algae [72,73].

The detectable sequence similarity observed between the *Partitiviridae* and *Amalgaviridae* also suggests that they share common ancestry [68], despite a wide range of hosts and genomic organizations. As such, it is important to determine whether amalgaviruses are restricted to plants [74,75,76] or, as the *Partitiviridae*, infect many divergent eukaryotic hosts such as fungi, plants and protists [77,78].

### 4.2. Divergent Homologs to Fungal Mitoviruses Detected in Ostreobium sp.

While we cannot formally identify host associations from our RNA-seq data alone, no fungal-associated contigs were detected in any of the libraries, strongly arguing against the mitovirus-like viruses detected in *Ostreobium* sp. as being fungal viruses. The presence of a potential new group of protist-associated mitoviruses is of importance as they have traditionally been viewed as restricted to fungal hosts and were only very recently identified in plants [79]. Similar to virus transfer between fungi and land plants, it is possible that the symbiosis and co-evolution between green algae and fungi [80,81] explains the close phylogenetic relationships of their viromes, perhaps including horizontal gene transfer events. Indeed, coral holobionts are the location of frequent interactions between endolithic algae, such as *Ostreobium* sp., and fungi [82,83,84]. Considering the high levels of sequence divergence between our viral sequences and those associated with fungi and within the clade formed by *Ostreobium* sp.-associated viruses, it seems likely that any such horizontal gene transfer events are not recent and may have occurred in Ulvophyceae or even Chlorophyta ancestors. It will be of considerable interest to examine this new group of mitoviruses across a larger set of green microalgae species, particularly whether their putative mitochondrial subcellular location is the result of an escape from cytoplasmic dsRNA silencing (as suggested for newly characterized plant mitoviruses [85]) or if they are relics of the eukaryotic endosymbiosis event, particularly as mitoviruses have bacterial counterparts—the *Leviviridae* [60]. More broadly, these newly-reported mitovirus-like sequences further illustrate the enormous diversity of hosts infected by the *Narnaviridae*, including such eukaryotic microorganisms as Apicomplexa, Excavates and Oomycetes hosts [86,87,88,89].

### 4.3. Detection of Plant Viruses in the Chlorarachniophytes

The apparent similarity between a Rhizarian (*C. reptans*) associated viral sequence and land-plant infecting viruses was striking. The Rhizaria and Archaeplastida are assumed to have diverged before the cyanobacteria primary endosymbiosis event, ca. 1.5 billion years ago [90,91]. Thus, a detectable sequence similarity between Rhizaria-associated viruses and those infecting land plants cannot be reasonably attributed to such an ancient evolutionary event. Rather, assuming these viruses actually infect *C. reptans*, the presence of such land plant-like viruses in Chlorarachniophyte would reflect their more recent acquisition in chlorarachniophytes through either horizontal transfer by common vector/symbiont/parasite ancestors or secondary endosymbiosis (eukaryote-to-eukaryote) events. Indeed, a secondary endosymbiosis event of a green alga in the core Chlorophyta, possibly related to Bryopsidales, led to the origin of the plastid of chlorarachniophytes between 578–318  Ma [33]. Whether this virus (i) constitutes a relic of viruses that infected this engulfed green algal endosymbiont, (ii) is part of the chlorarachniophyte cytoplasm or still associated with the periplastidial compartment (i.e., remnant cytoplasm of the endosymbiont), or (iii) interacts with the nucleomorph (remnants of the green algal endosymbiont nucleus) are key questions in the evolution of eukaryotic RNA viruses. While our data cannot provide answers and still require a formal virus-host association, it will be of interest to extend these analyses to euglenophytes and the dinoflagellate genus *Lepidodinium* where distinct secondary endosymbiosis with green algae have also occurred, as well as to cryptophytes that also contain the remnant nucleus of its red algae endosymbiont [92,93].

### 4.4. First Report of a Negative-Sense RNA Virus in Microalgae

Our detection of a *Bunyavirales*-like sequence in *C. reptans* is the first evidence of a negative-sense RNA virus in microalgae. Considering the extensive host range of the *Bunyavirales* (land plants, invertebrates, vertebrates and humans), their association with chlorarachniophyte hosts is plausible. However, given its very low abundance in *C. reptans*, additional work is clearly needed to retrieve the complete virus genome and to confirm the association of such bunya-like viruses with the chlorarachniophytes.

One of the greatest challenges in viral genomics is the ability to detect distant homologies, especially in rapidly evolving RNA viruses. As a first attempt to retrieve such ephemeral evolutionary signals, we scanned orphan contigs using RdRp protein structural information in addition to the standard primary amino acid sequence. Notably, both the RdRp profile and 3D protein structure comparison led to the identification of highly divergent RNA virus candidates, although these remain difficult to annotate. Efforts to better describe the repertoire of sequence and structure of viral RdRps are therefore central to unveiling the RNA virosphere in overlooked eukaryotic organisms.

## Figures and Tables

**Table 1:** Sample and library description.

Library	Species	Class/Family
ALG_1	*Kraftionema allantoideum*	Ulvophyceae/Kraftionemaceae
	*Microrhizoidea pickettheapsiorum*	Mamiellophyceae/Dolichomastigaceae
	*Dolichomastix tenuilepis*	Mamiellophyceae/Dolichomastigaceae
ALG_2	*Ostreobium sp. HV05007bc*	Ulvophyceae/Bryopsidales
ALG_3	*Chlorarachnion reptans*	Chlorarachniophyceae/Chlorarachnion
	*Lotharella sp*	Chlorarachniophyceae/Lotharella

**Table 2:** BLASTx results of the newly described virus-like sequences against nr database.

New Virus (Algal Host Species)	Length nt	PE Read Count (% Non rRNA)	BLASTx Hit GenBank Acc.	%ID	e-Value	BLASTx Hit Organism	BLASTx Hit Taxonomy
Amalga-like boulavirus (*K. allantoideum*)	1440	4313 (0.17%)	BAA25883	31	4.6 × 10^23^	BDRM	*Partitiviridae* (dsRNA)
Amalga-like chassivirus (*Ostreobium sp*.)	3399	1503 (0.02%)	BAA25883	28	1.8 × 10^38^	BDRM	*Partitiviridae* (dsRNA)
Amalga-like chaucrivirus (*Ostreobium sp*.)	4036	16,934 (0.23%)	BAA25883	33	3.3 × 10^103^	BDRM	*Partitiviridae* (dsRNA)
Amalga-like dominovirus (*Ostreobium sp*.)	4011	2996 (0.04%)	BAA25883	33	5.1 × 10^88^	BDRM	*Partitiviridae* (dsRNA)
Amalga-like lacheneauvirus (*Ostreobium sp*.)	3254	934 (0.01%)	BAA25883	27	3.5 × 10^39^	BDRM	*Partitiviridae* (dsRNA)
Partiti-like alassinovirus (*Ostreobium sp*.)	3658	5135 (0.07%)	BAB63954	29	1.6 × 10^48^	BDRC	Bryopsis cinicola * (dsRNA)
Partiti-like lacotivirus (*Ostreobium sp*.)	3273	92,840 (1.26%)	BAB63954	29	2.5 × 10^45^	BDRC	Bryopsis cinicola * (dsRNA)
Partiti-like adriusvirus (*Ostreobium sp*.)	4252	4833 (0.07%)	BAB63954	23	5.7 × 10^18^	BDRC	Bryopsis cinicola * (dsRNA)
Mito-like babylonusvirus (*Ostreobium sp*.)	2942	9029 (0.12%)	APG77166	38	9 × 10^42^	Shahe narna-like virus 6	Unclassified RNA virus (ssRNA)
Mito-like albercanusvirus (*Ostreobium sp*.)	2791	5294 (0.07%)	APG77166	39	7.2 × 10^41^	Shahe narna-like virus 6	Unclassified RNA virus (ssRNA)
Mito-like spartanusvirus (*Ostreobium sp*.)	2684	15,388 (0.21%)	ASM94099	38	2 × 10^32^	Barns Ness serrated wrack narna-like virus 3	*Narnaviridae* (ss+RNA)
Mito-like laruketanusvirus (*Ostreobium sp*.)	2928	14,185 (0.19%)	APG77166.1	36	5.8 × 10^33^	Shahe narna-like virus 6	Unclassified RNA virus (ssRNA)
Mito-like bobnusvirus (*Ostreobium sp*.)	2773	2621 (0.04%)	APG77166	34	6.1 × 10^40^	Shahe narna-like virus 6	Unclassified RNA virus
Mito-like picolinusvirus (*Ostreobium sp*.)	2714	8792 (0.12%)	YP 00228433	34	4.1 × 10^33^	Botrytis cinerea mitovirus 1	*Narnaviridae* (ss+RNA)
Mito-like bionusvirus (*Ostreobium sp*.)	3260	7529 (0.10%)	AXY40442	27	5.7 × 10^13^	Rhizophagus diaphanum mitovirus 1	*Narnaviridae* (ss+RNA)
Tombus-like chagrupourvirus (*Ostreobium sp*.)	3835	5418 (0.07%)	YP 009336735	36	5.1 × 10^45^	Hubei tombus-like virus 12	Unclassified RNA virus
Virga-like bellevillovirus (*C. reptans*)	2313	229 (0.01%)	AMO03254	29	4.4 × 10^44^	Boutonnet virus	Unclassified ssRNA virus (ssRNA)
Bunya-like bridouvirus (*C. reptans*)	2818	208 (0.01%)	APG79310	30	9.1 × 10^68^	Shahe bunya-like virus 1	Unclassified RNA virus

BDRM: Bryopsis mitochondria-associated dsRNA; BDRC: Bryopsis cinicola chloroplast-associated dsRNAs; * likely viral RdRp mis-annotated as a host protein.

**Table 3:** Results of the VOGdb and PFAM HMM analysis. Light pink: phage-like sequences. Grey: nonviral sequences; Light blue: DNA virus-like sequences; Orange: RNA virus-like sequences. Abund: expected read counts estimated using the RSEM program.

Contig Name	ORF	Abund.	Viral Hit	e-Value	Viral Hit Description	Viral-Like Hit Taxonomy	PFAM Hit ID	PFAM e-Value	PFAM Hit Description
ALG_2_DN19089_c0_g1_i1_len4252	ORF_1	4717	VOG03062	1.00 × 10^12^	REFSEQ hypothetical protein	Bacteriophage	-	-	-
ALG_2_DN19089_c0_g1_i1_len4252	ORF_1	4717	PF00680.20	2.70 × 10^5^	RNA dependent RNA polymerase	RdRP-1	-	-	-
ALG_2_DN19250_c2_g3_i5_len1869	ORF_1	743.91	VOG23558	1.00 × 10^4^	REFSEQ hypothetical protein	*Caudovirales; Siphoviridae*	-	-	-
ALG_2_DN18568_c0_g1_i1_len2977	ORF_1	511	VOG10478	1.30 × 10^6^	sp|Q05224|VG18 BPML5 Gene 18 protein	Bacteriophage	-	-	-
ALG_2_DN19013_c0_g1_i2_len1689	ORF_2	224.31	VOG22975	1.40 × 10^4^	REFSEQ carboxylesterase	*Caudovirales; Siphoviridae*	-	-	-
ALG_2_DN19410_c0_g2_i6_len1950	ORF_1	183.94	VOG12013	5.20 × 10^4^	sp|P03778|Y06 BPT7 Protein 0.6B	Viruses	PF16752.5	1.50 × 10^4^	Tubulin-specific chaperone C
ALG_2_DN18226_c0_g1_i1_len1259	ORF_1	157.61	VOG09820	2.90 × 10^4^	REFSEQ hypothetical protein	*Phycodnaviridae*; *Chlorovirus*	-	-	-
ALG_2_DN18993_c2_g2_i2_len2532	ORF_1	151.17	VOG08344	8.50 × 10^8^	REFSEQ hypothetical protein	Bacteriophage	PF13424.6	3.80 × 10^132^	Tetratricopeptide repeat
ALG_3_DN34624_c0_g1_i1_len2077	ORF_2	146	PF02123.16	8.50 × 10^5^	Viral RNA-directed RNA-polymerase	RdRP-4	-	-	-
ALG_2_DN18744_c0_g1_i3_len2080	ORF_1	134.99	VOG10472	4.10 × 10^4^	REFSEQ hypothetical protein	*Poxviridae*	-	-	-
ALG_2_DN19214_c2_g1_i7_len1432	ORF_2	118	VOG06927	6.90 × 10^4^	REFSEQ hypothetical protein	Bacteriophage	-	-	-
ALG_3_DN25592_c0_g1_i1_len1043	ORF_1	115	VOG01256	4.40 × 10^4^	sp|Q9QU29|ORF3 TTVB1 Uncharacterized ORF3 protein	dsDNA viruses	-	-	-
ALG_2_DN18451_c0_g1_i4_len2061	ORF_4	109.48	VOG17696	7.10 × 10^4^	REFSEQ hypothetical protein	Bacteriophage	PF16058.5	1.80 × 10^17^	Mucin-like
ALG_2_DN18451_c0_g1_i4_len2061	ORF_4	109.48	VOG17696	7.10 × 10^4^	REFSEQ_hypothetical_protein	Bacteriophage	PF16058.5	1.10 × 10^7^	Mucin-like
ALG_2_DN18732_c0_g2_i2_len2454	ORF_2	99.65	VOG09815	1.70 × 10^15^	REFSEQ hypothetical protein	*Phycodnaviridae*; *Chlorovirus*	-	-	-
ALG_2_DN18957_c0_g1_i1_len1408	ORF_2	97.98	VOG02199	8.40 × 10^4^	sp|Q5UR09|YR648 MIMIV Uncharacterized protein R648	*Ortervirales*	PF06156.13	4.70 × 10^4^	Initiation control protein YabA
ALG_2_DN18957_c0_g1_i2_len1657	ORF_2	89.04	VOG02199	3.50 × 10^4^	sp|Q5UR09|YR648 MIMIV Uncharacterized protein R648	*Ortervirales*	PF06156.13	2.20 × 10^4^	Initiation control protein YabA
ALG_2_DN19250_c2_g3_i3_len2203	ORF_1	71.48	VOG23558	7.80 × 10^5^	REFSEQ hypothetical protein	*Caudovirales*; *Siphoviridae*	-	-	-
ALG_2_DN19410_c0_g2_i7_len1634	ORF_1	49.51	VOG12013	9.90 × 10^4^	sp|P03778|Y06 BPT7 Protein 0.6B	Bacteriophage	-	-	-
ALG_2_DN11543_c0_g1_i1_len842	ORF_1	42	VOG20356	3.80 × 10^4^	REFSEQ hypothetical protein	*Caudovirales*; *Myoviridae*	-	-	-
ALG_2_DN19463_c5_g2_i1_len765	ORF_1	37.7	VOG24589	2.60 × 10^5^	REFSEQ hypothetical protein	*Caudovirales*; *Siphoviridae*	-	-	-
ALG_2_DN19174_c0_g2_i18_len938	ORF_1	36.39	VOG10625	9.70 × 10^4^	sp|Q05293|VG78 BPML5 Gene 78 protein	Bacteriophage	-	-	-
ALG_2_DN41289_c0_g1_i1_len750	ORF_1	36	VOG06662	9.50 × 10^5^	REFSEQ Cupin	Bacteriophage	-	-	-
ALG_2_DN22182_c0_g1_i1_len820	ORF_1	35	VOG02199	1.50 × 10^4^	sp|Q5UR09|YR648 MIMIV Uncharacterized protein R648	*Ortervirales*	PF06156.13	7.20 × 10^4^	Initiation control protein YabA
ALG_2_DN44027_c0_g1_i1_len815	ORF_1	33.98	VOG21678	3.70 × 10^4^	REFSEQ hypothetical protein	*Caudovirales*; *Myoviridae*	PF08614.11	6.00 × 10^5^	Autophagy protein 1(ATG16)
ALG_2_DN594_c0_g2_i1_len711	ORF_1	28	PF17501.2	2.80 × 10^4^	Viral RNA-directed RNA polymerase	Viral_RdRp_C	-	-	-
ALG_2_DN14271_c0_g1_i1_len772	ORF_1	25.12	VOG18617	2.20 × 10^4^	REFSEQ hypothetical protein	*Caudovirales*; *Siphoviridae*	PF13855.6	1.60 × 10^21^	Leucine rich repeat
ALG_2_DN18993_c2_g2_i1_len2178	ORF_1	21.18	VOG08344	4.70 × 10^7^	REFSEQ hypothetical protein	Bacteriophage	PF13374.6	1.40 × 10^126^	1Tetratricopeptide repeat
ALG_2_DN44027_c0_g1_i2_len783	ORF_1	19.02	VOG21678	3.60 × 10^4^	REFSEQ hypothetical protein	*Caudovirales*; *Myoviridae*	PF08614.11	7.70 × 10^5^	Autophagy protein 1(ATG16)
ALG_2_DN19463_c5_g2_i6_len928	ORF_1	14.34	VOG24589	6.40 × 10^5^	REFSEQ hypothetical protein	*Caudovirales*; *Siphoviridae*	-	-	-
ALG_2_DN19463_c5_g2_i4_len863	ORF_1	4.36	VOG24589	5.30 × 10^5^	REFSEQ hypothetical protein	*Caudovirales*; *Siphoviridae*	-	-	-

## Supplementary Materials

The following are available online at https://www.mdpi.com/1999-4915/12/10/1180/s1, Figure S1: Gel electrophoresis of RT-PCR to validate the putative new RNA viruses in each sample used for library construction and RNA-seq. Figure S2: MAFFT alignment of the HMM-detected RdRp candidates with Pfam RdRPp profiles. Figure S3: rRNA depletion and contig assembly results. Figure S4: Genomic organization of the ALG_2 Partiti-like sequences and Bryopsis mitochondria-associated dsRNA. Table S1: RNA-seq library preparation and sequencing results, Table S2: List of primers used in this study. Table S3 RdRp-based pairwise sequence identity of the viruses newly identified here: File S1: Krona graphs obtained using the KMA and CCMetagen methods for the ALG_1 library. File S2: Krona graphs obtained using the KMA and CCMetagen methods for the ALG_2 library. File S3: Krona graphs obtained using the KMA and CCMetagen methods for the ALG_3 library.
