# Supplementary material for: Metatranscriptomic Identification of Diverse and Divergent RNA Viruses in Green and Chlorarachniophyte Algae Cultures
Source: Viruses. 2020 Oct 19;12(10):1180. doi: 10.3390/v12101180 (PMC7594059; doi:10.3390/v12101180)
Supplement: Supplementary file 1 [file viruses-12-01180-s001.zip › Charon.File S3.html]

Javascript must be enabled to view this page.

magnitude
magnitudeUnassigned

ALG\_9.trinity.res

22795.64

21508.31

20655.88

20655.88
8844.22

11257.24
2761.46

8495.78

554.42
312.52

241.9

447.43

220.99

220.99

220.99

226.44

226.44

226.44

405

405

405

405

1069.53

401

401

401

401

401

668.53

668.53

368.65

368.65

368.65

299.88

299.88

299.88

217.8
