# Supplementary material for: Metatranscriptomic Identification of Diverse and Divergent RNA Viruses in Green and Chlorarachniophyte Algae Cultures
Source: Viruses. 2020 Oct 19;12(10):1180. doi: 10.3390/v12101180 (PMC7594059; doi:10.3390/v12101180)
Supplement: Supplementary file 1 [file viruses-12-01180-s001.zip › Charon.File S2.html]

Javascript must be enabled to view this page.

magnitude
magnitudeUnassigned

ALG\_2.trinity.res

234373.78

233193.37

201.14

201.14

201.14

201.14

201.14

201.14

201.14

1578.96

1578.96

1578.96

1578.96

1578.96

231413.27

231413.27

231413.27

231413.27

231413.27
97638.64

133774.63

662.31

240.57

421.74

421.74

421.74

421.74

421.74

421.74

518.1

518.1

518.1
