# Supplementary material for: Metatranscriptomic Identification of Diverse and Divergent RNA Viruses in Green and Chlorarachniophyte Algae Cultures
Source: Viruses. 2020 Oct 19;12(10):1180. doi: 10.3390/v12101180 (PMC7594059; doi:10.3390/v12101180)
Supplement: Supplementary file 1 [file viruses-12-01180-s001.zip › Charon.File S1.html]

Javascript must be enabled to view this page.

magnitude
magnitudeUnassigned

ALG\_1.trinity.res

3387.04

3387.04

442.38

226.38

226.38

226.38

226.38

216

216

216

216

277.5

277.5

277.5

277.5

2667.16

2667.16

1018.96

1018.96

1018.96

1018.96

1018.96

1648.2

1648.2
452.64

1195.56

1195.56

1195.56
