# Supplementary material for: Metatranscriptomic Identification of Diverse and Divergent RNA Viruses in Green and Chlorarachniophyte Algae Cultures
Source: Viruses. 2020 Oct 19;12(10):1180. doi: 10.3390/v12101180 (PMC7594059; doi:10.3390/v12101180)
Supplement: Supplementary file 1 [file viruses-12-01180-s001.zip › Charon.Supplementary Materials.Revised.pdf]

SUPPLEMENTARY MATERIALS

SUPPLEMENTARY FILES

File S1. Krona graphs obtained using the KMA and CCMetagen methods for the ALG_1 library. Contigs without any match in the nt database are not shown. Each circle represents a taxonomic level. Entries are grouped and coloured based on their lowest rank taxonomic classification.

File S2. Krona graphs obtained using the KMA and CCMetagen methods for the ALG_2 library. Contigs without any match in the nt database are not shown. Each circle represents a taxonomic level. Entries are grouped and coloured based on their lowest rank taxonomic classification.

File S3. Krona graphs obtained using the KMA and CCMetagen methods for the ALG_3 library. Contigs without any match in the nt database are not shown. Each circle represents a taxonomic level. Entries are grouped and coloured based on their lowest rank taxonomic classification.

SUPPLEMENTARY FIGURES

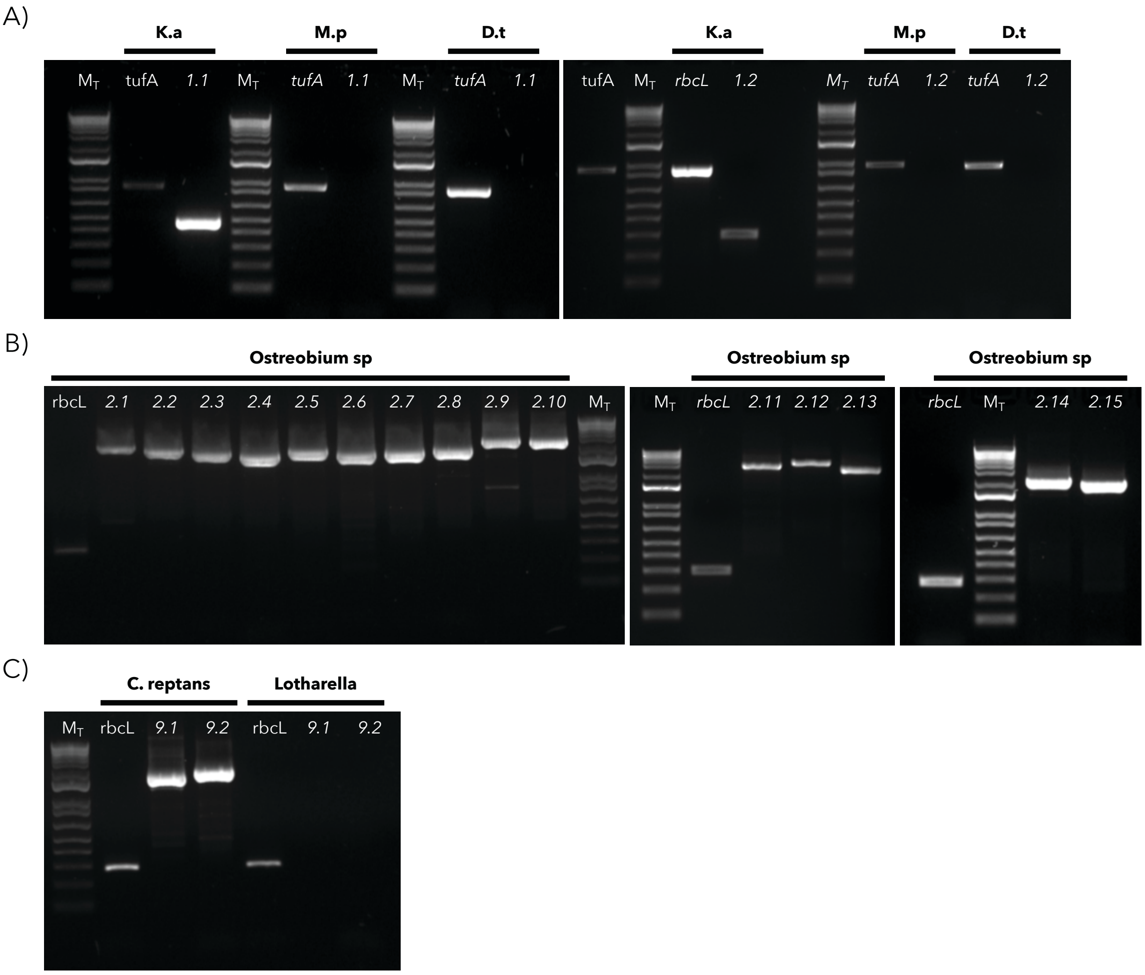

Figure S1. Gel electrophoresis of RT-PCR to validate the putative new RNA viruses in each sample used for library construction and RNA-seq. RT-PCR validation in (A) ALG_1; (B) ALG_2; and (**C**) ALG_3. rbcL: ribulose-bisphosphate carboxylase gene, tufA: elongation factor Tu, both used as positive PCR-controls. K.a: *Kraftionema allantoideum*; M.p: *Microrhizoidea pickettheapsiorum* ; D.t :*Dolichomastix tenuilepsis*. 1-9: virus tested (refer to Table S2 for virus name correspondences).

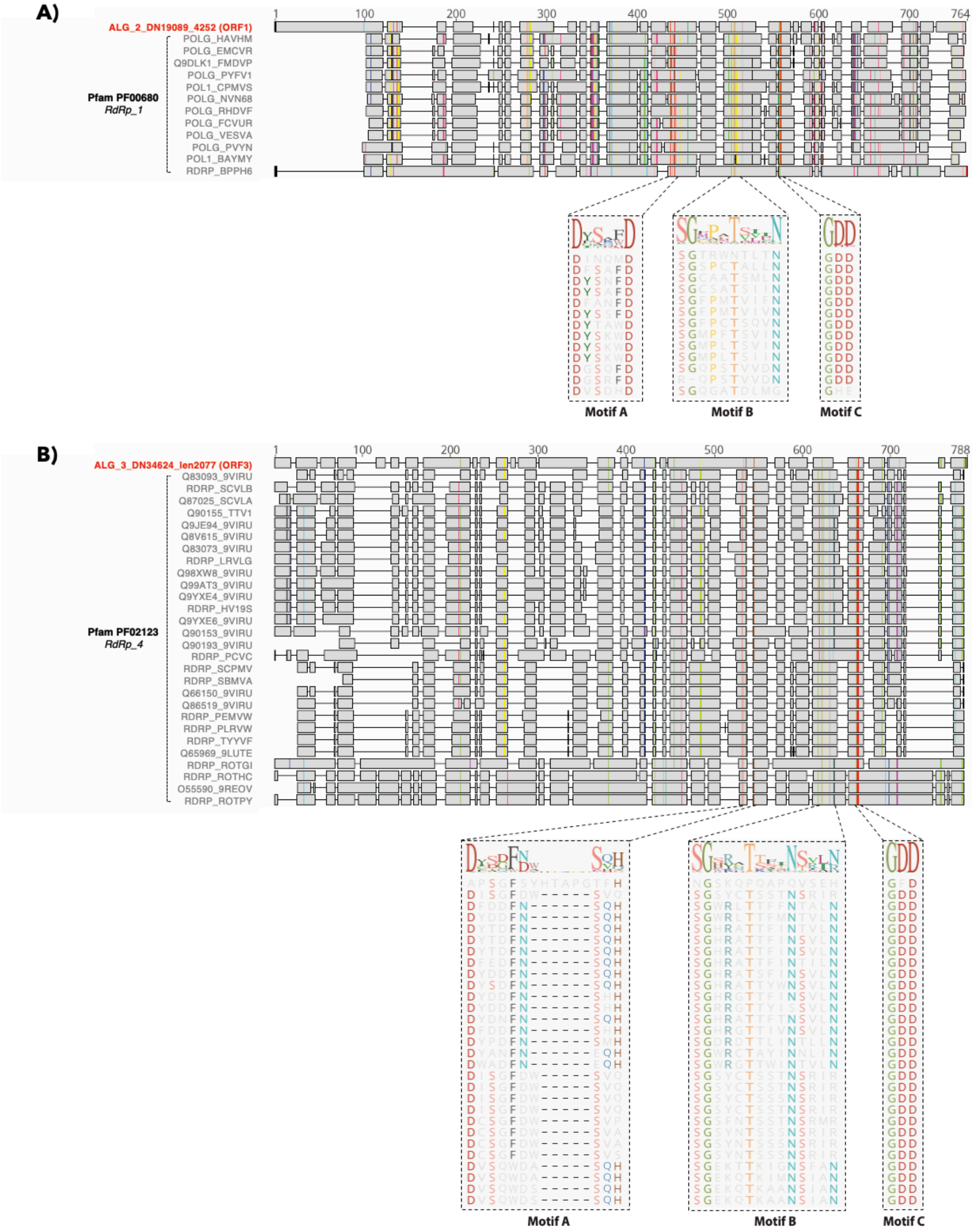

Figure S2. MAFFT alignment of the HMM-detected RdRp candidates with Pfam RdRPp profiles. (A) Alignment of the ALG_2_DN19089-encoded ORF with the PFAM RdRp_1 profile entries. (B) ALG_3_DN34624-encoded ORF with the PFAM RdRp_4 profile entries. Coloured boxes represent the agreement with consensus. The dash boxes highlight the sequence logo and sequence alignment obtained at the three most conserved RdRp functional motifs. UniprotKB locus accessions of each RdRp used in the Pfam profiles are indicated on the left part of the alignments.

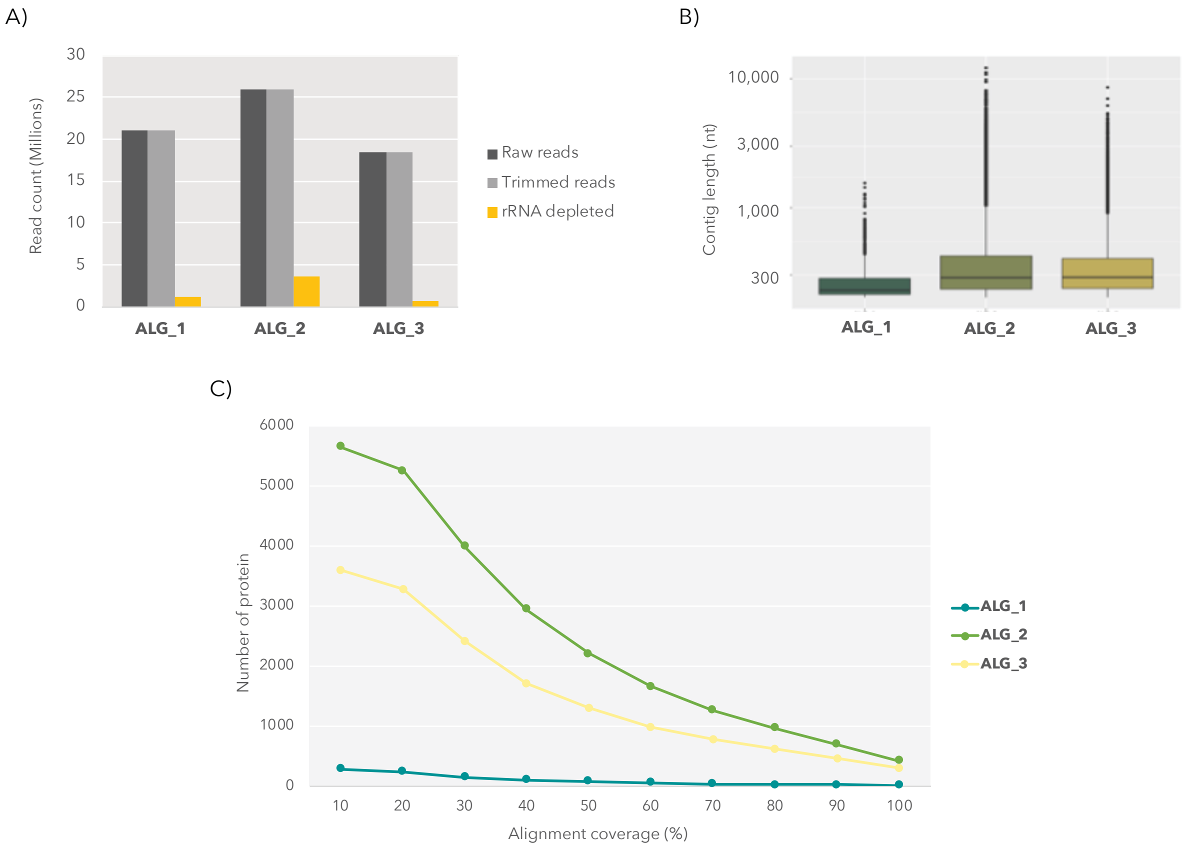

Figure S3. rRNA depletion and contig assembly results. (A) Read counts before and after trimming and SortmeRNA rRNA depletion. (B) Trinity assembly quality expressed as the average length of contig assembled for each library. (C) Comparison of full-length transcripts obtained in each library. Dots correspond to the number of top matching SwissProt database entries with coverage equal or higher than the x-value when align to library transcripts (corresponding to the ">bin_below" value obtained with "analyze_blastPlus_topHit_coverage.pl" script available in the Trinity v2.5.1 package.

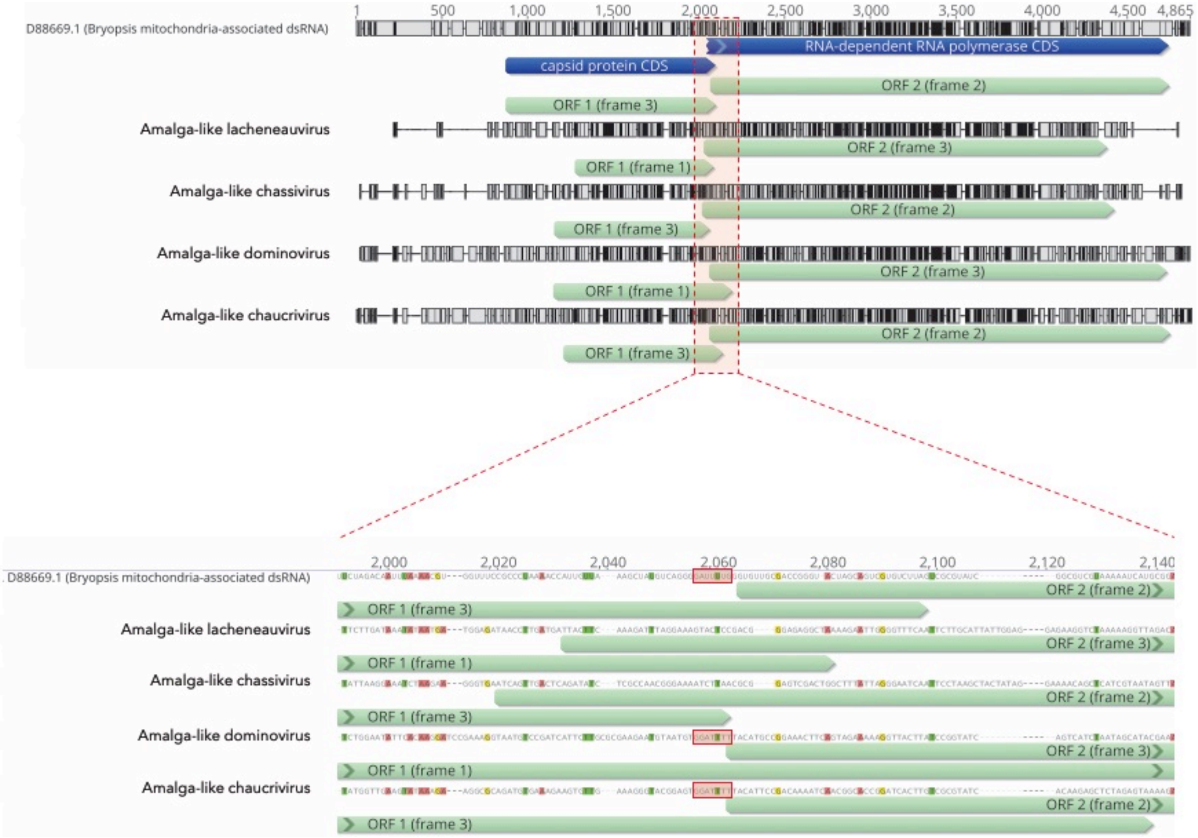

Figure S4. Genomic organization of the ALG_2 Partiti-like sequences and Bryopsis mitochondria-associated dsRNA. ORFs are predicted using the mold protozoan mitochondrial genetic code (translation table 4). Ribosomal -1 frameshifting motifs “GGAUUUU” are indicated in red boxes. Grey and black blocks: disagreement and agreement to the consensus sequence, respectively.

SUPPLEMENTARY TABLES

Table S1. RNA-seq library preparation and sequencing results.

Library ID	Library prep protocol	Nb paired-end reads	Data yield (bp)
**ALG_1**	SMARTer Stranded Total RNA-Seq Kit v2 Pico Input Mammalian	21,014,207	5.30 Gb
**ALG_2**	TruSeq stranded/RiboZero gold	25,963,002	6.54 Gb
**ALG_3**	SMARTer Stranded Total RNA-Seq Kit v2 Pico Input Mammalian	18,448,371	4.65 Gb

Table S2. List of primers used in this study. tufA primers have been retrieved from ref.[90]. Ta: annealing temperature used in corresponding PCR reactions.

Sequence ID	Primer ID	Sequence	Direction	Ta (°C)	Product Size
**Hypothetical amalga-like (ALG_1_DN2506)**	1.1F	CATTGCGTGAGTGGTATGCG	forward	63	867
	1.1R	GTGCGCCTCTTAGCTATTGT	reverse		
**Amalga-like boulavirus**	1.2F	CGCTCATTGCACTAAAGATGACA	forward	62	1112
	1.2R	TCAACCGTGTAAGGAGTTTGA	reverse		
**Amalga-like chassivirus**	2.1F	GCTGCCGCTCACAATAGAGA	forward	65	2616
	2.1R	GGGTAACGGGGTCCTGTATG	reverse		
**Tombus-like chagrupourvirus**	2.2F	CCAGCCGATTGATCTCGACA	forward	65	2360
	2.2R	ATGGACTTGTAATGGGCGCA	reverse		
**Amalga-like chaucrivirus**	2.3F	GATTGACGCAGAACACGACG	forward	65	2105
	2.3R	ACAGACTCCGCCTCGAGTAA	reverse		
**Mito-like babylonusvirus**	2.4F	CCTGAGGGCACTTCTGTGTT	forward	65	2000
	2.4R	CCATCATACGTTGGCGGAGA	reverse		
**Mito-like bionusvirus**	2.5F	AGCAGAAGGAGGTCTCGCTA	forward	65	2311
	2.5R	TTTCGCTAAGGGGAACGACC	reverse		
**Mito-like spartanusvirus**	2.6F	GATCGCCTCCGTCTACATGG	forward	65	2032
	2.6R	TTTGGACAGTCGGTACCGTC	reverse		
**Mito-like laruketanusvirus**	2.7R	TGCAAGCTTCCCACCTTGAT	reverse	65	2070
	2.7F	GGAAAATCGCCAAAGCAGGG	forward		
**Mito-like bobnusvirus**	2.8R	GGCCTTTTAGAAAGCCAGCG	reverse	65	2154
	2.8F	CCGGATGCCTCTTACGTGAA	forward		
**Amalga-like dominovirus**	2.9F	GTTGCTGAGGGTCTTGTCGA	forward	65	2809
	2.9R	ACGATTTTCGAGACGCCTGT	reverse		

**Amalga-like lacheneauvirus**	2.10F	CTGGCAGCTACACACGAGAA	forward	65	2675
	2.10R	GCGAATCAGTTGTTCCCGGA	reverse		
**Partiti-like alassinovirus**	2.11F	GGATGCGACCGTTGAATGC	forward	65	2867
	2.11R	TCACTAGGGCACTCCACGA	reverse		
**Partiti-like adriusvirus**	2.12F	GGTTTGAACGCGGGGTAGA	forward	62	3357
	2.12R	TCGAATTCATCAGTGGGCA	reverse		
**Partiti-like lacotivirus**	2.13F	TTTCAATGGCGGCGAGTCA	forward	65	2503
	2.13R	CGGCCATATCTTCATCGTCCA	reverse		
**Mito-like picolinusvirus**	2.14F	CAGAACGTTGTTCCGGCTTG	forward	65	2114
	2.14R	TCCCCGAACGTAAGGATCCT	reverse		
**Mito-like albercanusvirus**	2.15F	CCGCGGCCCAATCAAATATG	forward	65	1973
	2.15R	CTGGGTAAGCACGCATTTCG	reverse		
**Virga-like bellevillovirus**	9.1F	AAGTTGTGTGATAGGGGGCG	forward	65	1741
	9.1R	TAAACATCCACACGCCGTCA	reverse		
**Bunya-like bridouvirus**	9.2F	TGGCTGCACTAACCCTCATG	forward	65	2049
	9.2R	GCTGTGTTGCTTGCATCCAA	reverse		
**rbcL**	rbcL_F	CGTGCDATGCAYGCNGTWAT	forward	63	275
	rbcL_R	TGCCAWACRTGAATACCACC	reverse		
**tufA**	tufA_F	GGNGCNGCNCAAATGGAYGG	forward	55	758-901
	tufA_R	CCTTCNCGAATMGCRAAWCGC	reverse		

Table S3. RdRp-based pairwise sequence identity of the viruses newly identified here. Top: Amalga-like RdRp; Middle: BDRC-like RdRp; Bottom: Mitovirus-like RdRp.

Amalga-like RdRp	Amalga-like chassivirus	Amalga-like lacheneauvirus	Amalga-like chaucrivirus	Amalga-like dominovirus
Amalga-like boulavirus	19	21	16	16
Amalga-like chassivirus	-	47	22	20
Amalga-like lacheneauvirus		-	22	21
Amalga-like chaucrivirus			-	36

BDRC-like RdRp	Partiti-like alassinovirus	Partiti-like lacotivirus
Partiti-like adriusvirus	20	20
Partiti-like alassinovirus	-	32

Mitovirus-like RdRp	Mito-like picolinus virus	Mito-like babylonus virus	Mito-like albercanus virus	Mito-like laruketanus virus	Mito-like bobnus virus	Mito-like bionusvirus
Mito-like spartanusvirus	46	19	21	21	20	15
Mito-like picolinusvirus	-	17	20	19	19	13
Mito-like babylonusvirus		-	22	22	20	14
Mito-like albercanusvirus			-	26	25	16
Mito-like laruketanusvirus				-	37	14
Mito-like bobnusvirus					-	12
